# Splenic Infarction as a Complication of Acute Epstein-Barr Virus Infection in an 18-Year-Old Woman

**DOI:** 10.7759/cureus.107816

**Published:** 2026-04-27

**Authors:** Prabesh Angbuhang, Ahmed Amer, Anna Vaulks, Sajjad Pathan, Al Motasim Bella Abu Laban

**Affiliations:** 1 Emergency Department, Royal Surrey County Hospital, Guildford, GBR; 2 Medicine and Surgery Department, Royal Surrey County Hospital, Guildford, GBR

**Keywords:** conservative management, epstein-barr virus, glandular fever, infectious mononucleosis, splenic infarction, splenomegaly, young adult

## Abstract

Infectious mononucleosis, caused by Epstein-Barr virus (EBV), is a common illness in adolescents and young adults that usually follows a benign, self-limiting course. We report the case of an otherwise healthy 18-year-old woman who developed a radiologically confirmed splenic infarct presenting with left upper quadrant abdominal pain as a rare complication of infectious mononucleosis. Imaging demonstrated splenomegaly with a subcapsular wedge-shaped splenic infarct. She was managed conservatively with analgesia, observation, activity restriction, and interval imaging, with complete radiological and clinical resolution. This case highlights splenic infarction as a rare but likely under-recognised complication of EBV infection that can mimic more common abdominal pathology. Clinicians should maintain a high index of suspicion for splenic complications in patients with infectious mononucleosis who develop new or focal left upper quadrant abdominal pain, as prompt diagnosis facilitates appropriate management.

## Introduction

Infectious mononucleosis is most commonly caused by primary Epstein-Barr virus (EBV) infection and typically presents with fever, pharyngitis, lymphadenopathy, lymphocytosis, and transient derangement of liver function tests [1,2]. The incidence of splenic infarction in EBV infectious mononucleosis is unknown; evidence is largely limited to case reports and case-based systematic reviews. A 2023 systematic literature review identified 29 published cases of EBV-associated splenic infarction compared with 186 splenic ruptures over the period 1970-2022 [3]. The true incidence of EBV-associated splenic infarction likely exceeds published case numbers, as many cases may remain undiagnosed if abdominal imaging is not obtained for cases presenting with left upper quadrant abdominal pain in infectious mononucleosis patients, particularly in non-specialist settings [4].

Spontaneous or trauma-related splenic rupture is the most feared complication of infectious mononucleosis, with an estimated incidence of 0.1-0.5% of cases [5,6]. In contrast, splenic infarction appears to be exceedingly rare, with only a few dozen cases described in the literature despite the very high global incidence of EBV infection [4]. A systematic review of EBV-associated splenic complications identified far more cases of splenic rupture than infarction, suggesting that splenic infarction remains an under-recognised and under-reported entity [3]. Interestingly, recent literature suggests that male predominance occurs in both conditions, with 70% of infarction cases occurring in males [3]. 

We report the case of an 18-year-old woman with acute EBV infectious mononucleosis who presented to a UK emergency department with left upper quadrant abdominal pain, was found to have hepatosplenomegaly and a wedge-shaped splenic infarct on CT, and was successfully managed conservatively with complete sonographic resolution. This case exemplifies the clinical challenge that EBV-associated splenic infarction may present atypically, with abdominal pain as the sentinel symptom in the absence of prominent fever, pharyngitis, or lymphadenopathy, potentially delaying diagnosis.

## Case presentation

An 18-year-old woman with no significant past medical history presented to the emergency department in the UK with abdominal pain. One week earlier, she had returned from Turkey, where she had been hospitalised for three days with left upper quadrant abdominal pain, vomiting, and a presumed gastroenteritis. At the Turkish hospital, she was reported to have deranged liver function tests, raised inflammatory markers, and an abdominal ultrasound showing splenomegaly. She was treated with intravenous fluids, cefazolin, metoclopramide, and hyoscine butylbromide, with symptomatic improvement, and was discharged home.

On presentation to the UK emergency department, she described a two-week history of upper respiratory tract symptoms that had progressed from a dry cough to a yellow productive cough. She denied fever, dysuria, bowel disturbance, or trauma. There was no personal or family history of thromboembolism, congenital heart disease, valvular disease, haematological disorders, or autoimmune disease. She was a non-smoker and was not taking hormonal contraception or any regular medications.

**Figure 1:**
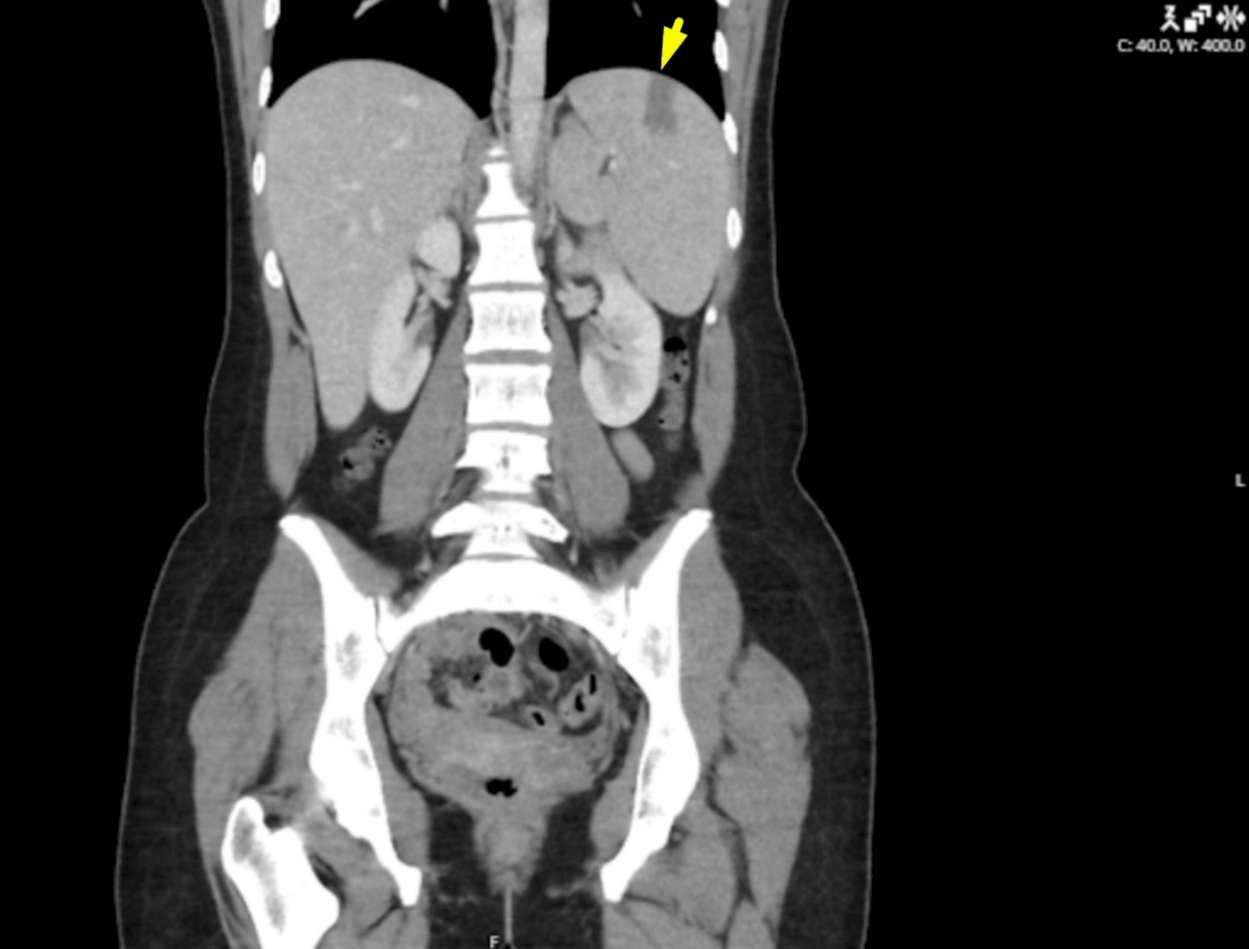
Coronal CT image of the abdomen Splenic infarct visualised on coronal view (indicated by arrow).

On arrival at the emergency department, her observations were within normal limits. The temperature was 36.6°C, heart rate 95 beats per minute, respiratory rate 17 breaths per minute, blood pressure 107/73 mmHg, and oxygen saturation 97% on room air. Examination of the oropharynx showed mild tonsillar exudate without significant enlargement or airway compromise. There was no cervical lymphadenopathy. Chest auscultation was clear. Cardiovascular examination was unremarkable. Abdominal examination revealed localised tenderness in the left upper quadrant area without signs of peritonism and no palpable masses. Peripheral examination showed no oedema, no stigmata of chronic disease, and no features of autoimmune pathology (Figure 1).

Initial investigations included a normal chest radiograph and routine blood tests. Full blood count demonstrated lymphocytosis with pleomorphic atypical lymphocytes on blood film, consistent with reactive lymphocytes seen in infectious mononucleosis. Liver function tests showed mildly deranged liver functions consistent with acute EBV-associated hepatitis. Renal function was normal. C-reactive protein was mildly elevated. A heterophile antibody ("glandular fever") screen was positive, supporting a diagnosis of acute EBV infectious mononucleosis (Table 1). 

**Table 1:** Laboratory investigations on presentation (H) indicates value above reference range. eGFR: estimated glomerular filtration rate

Investigation	Result	Reference range	Units
Biochemistry			
Sodium	138	135-145	mmol/L
Potassium	4.6	3.5-5.3	mmol/L
Urea	4.3	2.5-6.8	mmol/L
Creatinine	66	43-74	µmol/L
eGFR	>90	>90	mL/min/1.73 m²
Albumin	40	35-49	g/L
Total bilirubin	11	0-20	µmol/L
Alkaline phosphatase	114 (H)	48-95	U/L
Alanine transaminase	118 (H)	8-24	U/L
C-reactive protein	30 (H)	0-5	mg/L
Haematology			
White cell count	10.4	4.0-11.0	×10⁹/L
Haemoglobin	147	115-165	g/L
Platelet count	235	150-450	×10⁹/L
Red cell count	4.67	3.5-5.5	×10¹²/L
Haematocrit	0.435	0.37-0.47	L/L
Mean cell volume	93.2	75-105	fL
Mean cell haemoglobin	31.5	26-35	pg
Neutrophils	4.1	2.0-7.5	×10⁹/L
Lymphocytes	5.5 (H)	0.8-4.0	×10⁹/L
Monocytes	0.8	0.2-1.3	×10⁹/L
Eosinophils	0.0	0.0-0.4	×10⁹/L
Basophils	0.1	0.0-0.1	×10⁹/L
Blood film			
Film comment	Pleomorphic lymphocytosis with some large lymphocytes with low nuclear cytoplasmic ratio with variable nuclear and cytoplasmic outlines, some were noted to be scalloping red cells. Basophilic cytoplasm noted in some cells with others has basophilia predominantly at the cytoplasmic margins. Film in keeping with glandular fever		
Urinalysis (midstream urine)			
Glucose	Negative	Negative	-
Ketones	Trace	Negative	-
Specific gravity	1.030	1.005-1.030	-
Protein	Negative	Negative	-
Nitrites	Negative	Negative	-
Leucocytes	+	Negative	-
Blood	+	Negative	-
Urine pregnancy test	Negative	Negative	-
Serology			
Glandular fever screen (heterophile antibody)	Positive	Negative	-

A focused bedside abdominal ultrasound performed in the emergency department demonstrated splenomegaly without free intra-abdominal fluid or capsular disruption. No definite focal splenic lesion was initially reported. Given the absence of peritonism and stable observations, she was discharged with advice to avoid contact sports, and a formal ultrasound was arranged for the following day.

**Figure 2:**
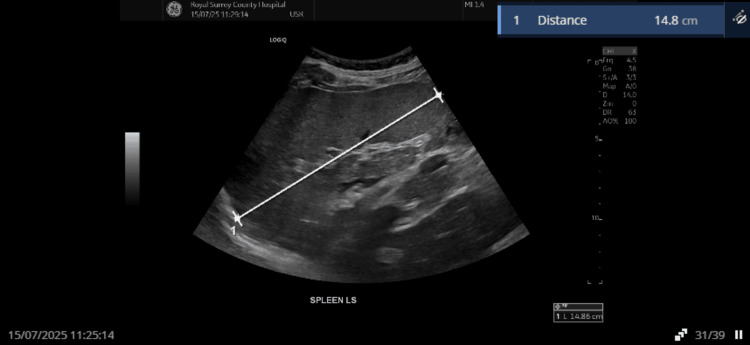
Splenomegaly detected on the next-day abdominal ultrasound scan Abdominal ultrasound demonstrating splenomegaly (14.8 cm) initially raising concern for infarct, haematoma, or other pathology.

The next day, an official comprehensive abdominal ultrasound performed by a sonographer confirmed splenomegaly (14.8 cm) with a 42 × 29 × 39 mm echopoor lesion in the posterior spleen, reported as possible haematoma, infarct, or lesion, and recommending cross-sectional imaging (Figure 2).

**Figure 3:**
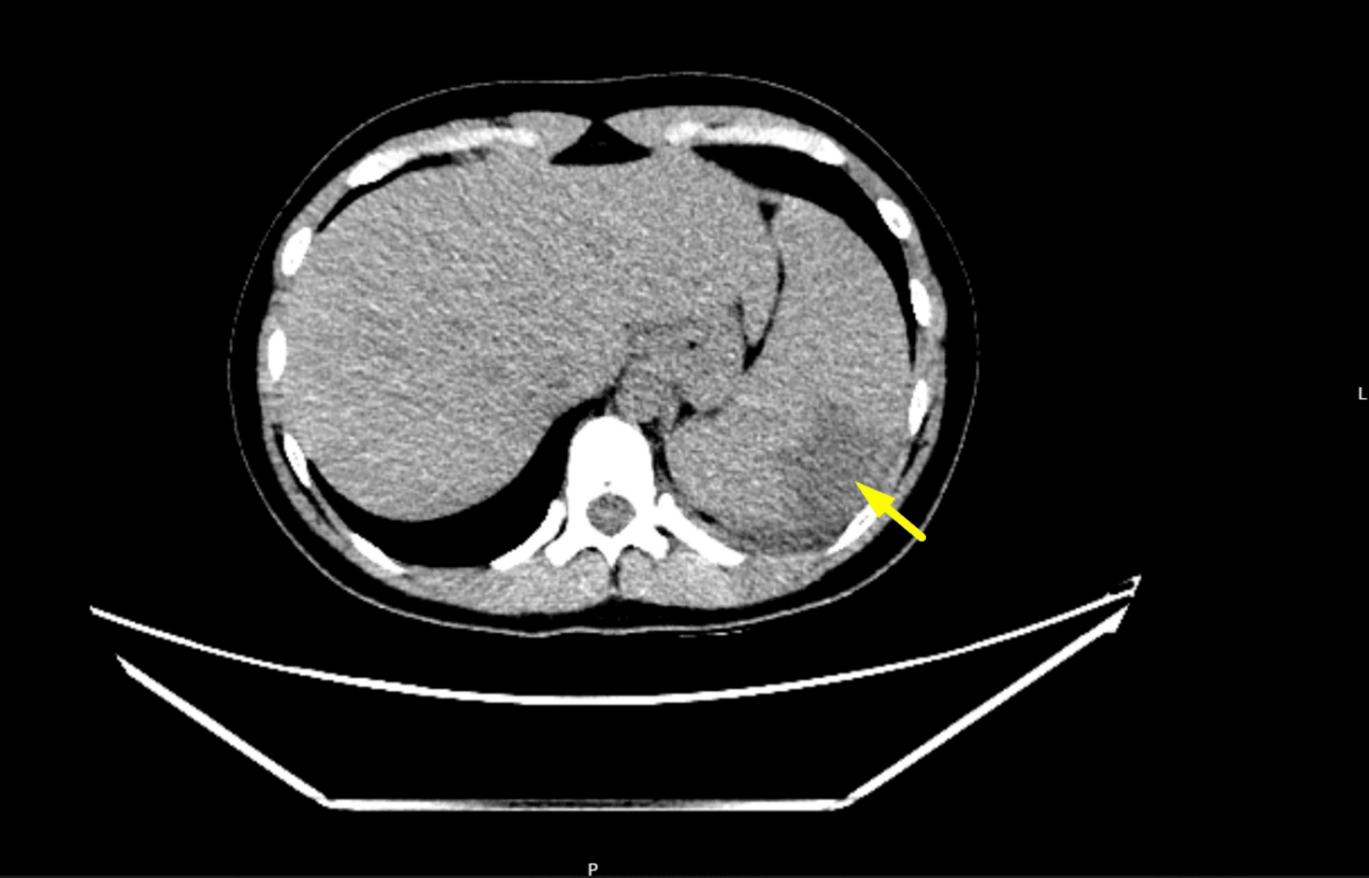
Cross-sectional contrast-enhanced CT of the abdomen Cross-sectional contrast-enhanced CT of the abdomen (portal venous phase) showing splenomegaly (13.3 cm) with a characteristic subcapsular peripheral wedge-shaped hypoenhancing area along the splenic dome (indicated by arrow), consistent with splenic infarction.

**Figure 4:**
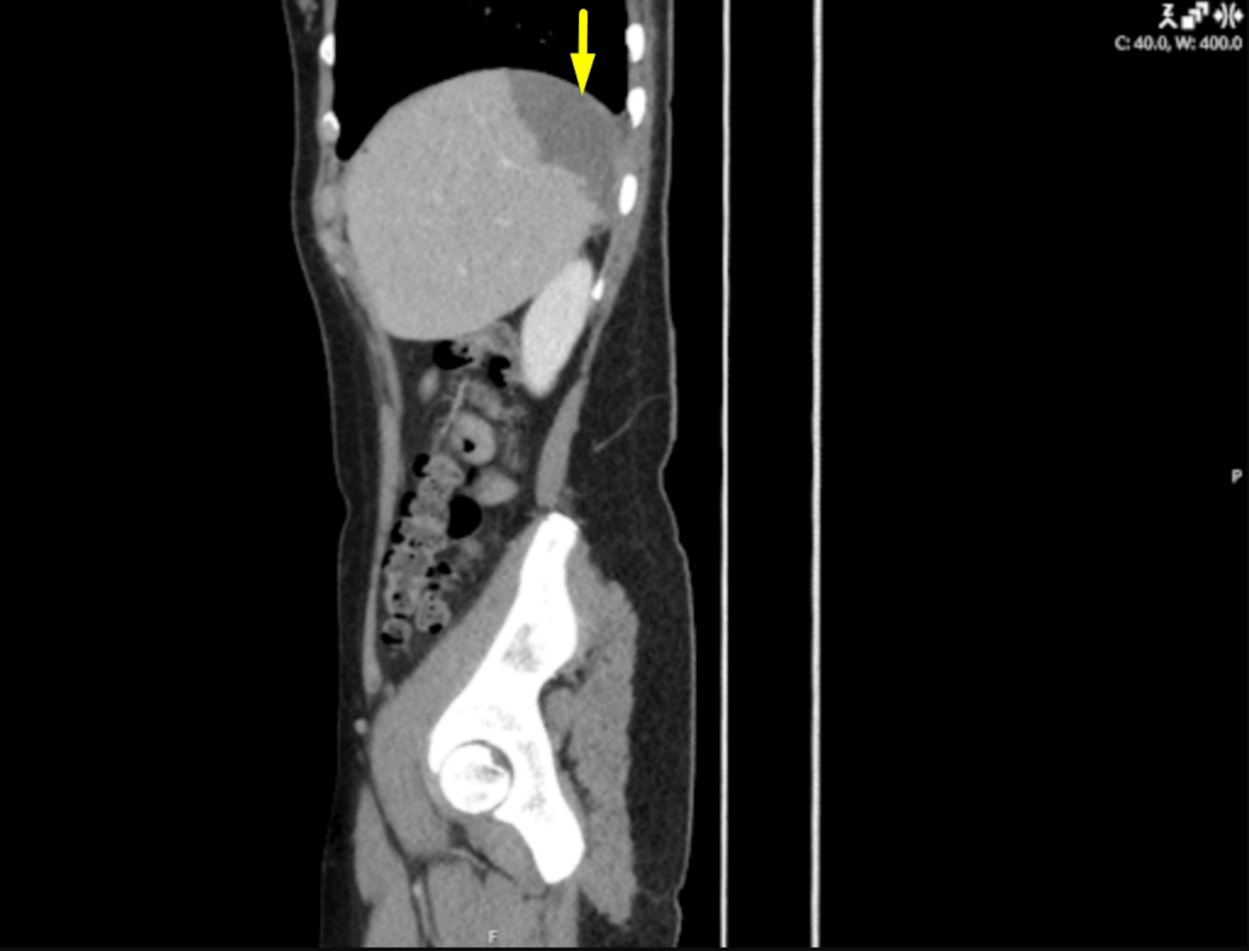
Sagittal CT image of the abdomen Subcapsular wedge-shaped hypoenhancing area at the splenic dome, consistent with an infarct (indicated by arrow).

Contrast-enhanced CT of the abdomen and pelvis demonstrated mild hepatosplenomegaly, with the spleen measuring 13.3 cm craniocaudally, and revealed a subcapsular peripheral wedge-shaped hypoenhancing area along the splenic dome, in keeping with an infarct. There was no evidence of subcapsular haematoma, rupture, free fluid, or additional abdominal pathology. Renal function remained normal, and inflammatory markers stayed mildly elevated (Figure 3 and Figure 4).

Multidisciplinary team input was obtained. The case was discussed with general surgery and haematology. In view of the patient's stable haemodynamics, no evidence of active bleeding, no major cardioembolic or haematological risk factors, and the likely inflammatory-hypercoagulable mechanism secondary to acute EBV, a conservative approach was agreed. This included analgesia, 24-hour in-hospital observation, and a follow-up ultrasound at six weeks.

**Figure 5:**
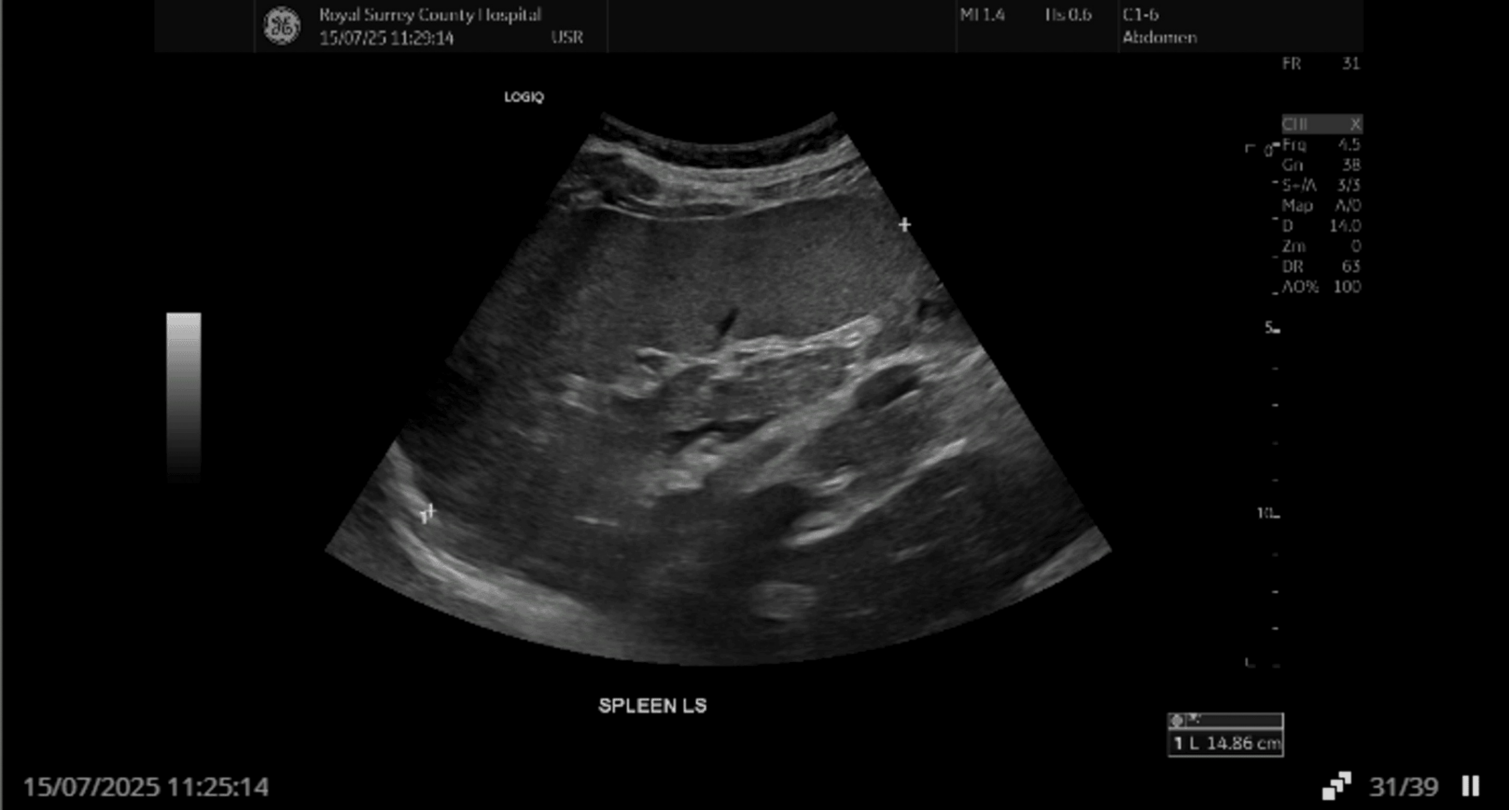
Follow-up abdominal ultrasound Four months post-presentation, ultrasound imaging demonstrating complete resolution of the splenic infarct. The spleen now measures 9.38 cm with normal homogeneous echotexture and no focal lesion visible.

Follow-up abdominal ultrasound performed at approximately four months post-presentation demonstrated complete resolution of the splenic infarct. The spleen measured 9.38 cm with normal homogeneous echotexture and no focal lesion visible. The liver, pancreas, gallbladder, biliary tree, and both kidneys demonstrated normal sonographic appearances. Clinically, the patient reported full symptom resolution (Figure 5).

## Discussion

Our patient's presentation aligns with the typical demographic profile described in the literature. A systematic review by Heo et al. reported a median age of 23.5 years (range 7-57 years) for EBV-associated splenic infarction, with most cases occurring in otherwise healthy individuals [7]. Left upper quadrant pain was the most common presenting symptom, observed in 79% of reported cases, consistent with our patient's chief complaint [7]. The median time from onset of infectious mononucleosis symptoms to diagnosis of splenic infarction has been reported as five days (range 1-25 days) [7]. Ultrasound is a useful first-line tool in assessing splenomegaly and can detect larger infarcts. However, contrast-enhanced CT remains the gold standard for demonstrating the classic peripheral wedge-shaped hypoattenuating appearance of a splenic infarct and for excluding haematoma, abscess, rupture, or malignancy [8,9]. In this case, ultrasound detected splenomegaly with a focal echopoor lesion, whilst CT confirmed the wedge-shaped infarct consistent with previously reported descriptions [8]. When assessed by ultrasound imaging, splenomegaly is far more common than clinical examination suggests. A landmark ultrasound study of 29 hospitalised patients with infectious mononucleosis demonstrated that all patients had an enlarged spleen on ultrasound scanning. However, only 17% of these enlarged spleens were palpable on physical examination, highlighting the significant limitation of clinical assessment alone [10]. This finding underscores the critical importance of abdominal ultrasound in all patients with confirmed infectious mononucleosis.

This case demonstrates that splenic infarction should be considered in the differential diagnosis of EBV-associated abdominal pain, as misattribution to other causes can delay diagnosis and appropriate imaging [7]. Splenic infarction in adults is generally uncommon and is most frequently attributed to embolic heart disease, haematological malignancy, myeloproliferative neoplasms, haemoglobinopathies, hypercoagulable states, and systemic infection [11,12]. Large series and systematic reviews demonstrate that emboligenic heart disease and haematological disorders account for a substantial proportion of cases, with infection forming a less common but important aetiological group [11,12]. Within this infectious subgroup, EBV-associated infectious mononucleosis is recognised as a rare cause of splenic infarction, usually in young, otherwise healthy individuals [4,13]. Reports suggest that EBV-related splenic infarction may be more common than previously thought but may go undetected if abdominal imaging is not pursued in cases of left upper abdominal pain during infectious mononucleosis [4]. One of the pathophysiologies for spleen infarct is that during the acute phase of EBV infection, there is believed to be an increased thrombotic tendency that predisposes patients to splenic infarction [4].

Management of EBV-related splenic infarction is generally conservative and tailored to the clinical presentation. Most cases in the literature were managed with observation, analgesia, hydration, and avoidance of abdominal trauma, with excellent outcomes [3,7]. In the systematic review by Toti et al., all cases of splenic infarction were managed conservatively with no fatalities, contrasting with the 4.8% mortality rate observed in splenic rupture cases [3]. Activity restriction is essential to reduce the risk of rupture during splenic healing. Current guidelines recommend avoiding contact sports and strenuous activity for at least three to four weeks, though some evidence suggests longer periods of eight weeks or until resolution of splenomegaly [1,14]. However, in the systematic review of Toti et al., approximately 80% (n = 139) of splenic rupture cases occurred within three weeks of the onset of mononucleosis symptoms [3]. Activity restrictions should be guided by interval imaging and clinical improvement rather than fixed timeframes. In this case, the patient achieved complete imaging resolution by four months, consistent with the expected timeline for radiological resolution of splenic infarction [7].

The role of anticoagulation in EBV-associated splenic infarction remains debated and is usually reserved for patients with additional thrombotic risk factors or evidence of progressive thrombosis [3]. Unlike thromboembolism in other settings, EBV-related splenic infarction typically does not benefit from anticoagulation. This approach is supported by Heo et al. who found that 74% of patients managed medically without anticoagulation achieved complete resolution, and Toti et al.'s systematic review found no fatal outcomes in conservatively managed infarction cases despite no anticoagulation use [3,7]. In this case, haematology consultation recommended against anticoagulation, consistent with the approach described in most published evidence. Given the transient nature of the hypercoagulable state and the generally favourable outcomes with supportive care alone, therapeutic anticoagulation appears unnecessary in most cases.

Splenectomy in EBV-associated splenic infarction is increasingly reserved for haemodynamic instability or failure of conservative management [3]. The complete resolution of splenomegaly and infarction on follow-up imaging at four months is consistent with the expected natural history of this complication [3,7].

## Conclusions

This case describes a rare occurrence of EBV-associated splenic infarction in an otherwise healthy 18-year-old woman presenting with left upper quadrant abdominal pain, lymphocytosis, and deranged liver function tests. Further investigation with imaging was performed with ultrasound which showed splenomegaly and a CT-confirmed wedge-shaped splenic infarct. Full radiological resolution under conservative management was achieved. It is essential to differentiate it from splenic rupture, which remains the more commonly feared complication, as both may present with left upper quadrant pain. Timely imaging is critical to establish an accurate diagnosis and guide appropriate management. A focused work-up, including early ultrasound or CT, should be considered to identify any potential splenic pathology and prevent its complications. In stable patients without signs of active bleeding or high-risk features, conservative management with analgesia, observation, avoidance of strenuous activity, and follow-up imaging can lead to full recovery, as demonstrated in this case.
